# Complete genome sequence of the extremely halophilic *Halanaerobium praevalens* type strain (GSL^T^)

**DOI:** 10.4056/sigs.1824509

**Published:** 2011-06-30

**Authors:** Natalia Ivanova, Johannes Sikorski, Olga Chertkov, Matt Nolan, Susan Lucas, Nancy Hammon, Shweta Deshpande, Jan-Fang Cheng, Roxanne Tapia, Cliff Han, Lynne Goodwin, Sam Pitluck, Marcel Huntemann, Konstantinos Liolios, Ioanna Pagani, Konstantinos Mavromatis, Galina Ovchinikova, Amrita Pati, Amy Chen, Krishna Palaniappan, Miriam Land, Loren Hauser, Evelyne-Marie Brambilla, K. Palani Kannan, Manfred Rohde, Brian J. Tindall, Markus Göker, John C. Detter, Tanja Woyke, James Bristow, Jonathan A. Eisen, Victor Markowitz, Philip Hugenholtz, Nikos C. Kyrpides, Hans-Peter Klenk, Alla Lapidus

**Affiliations:** 1 DOE Joint Genome Institute, Walnut Creek, California, USA; 2 DSMZ - German Collection of Microorganisms and Cell Cultures GmbH, Braunschweig, Germany; 3 Los Alamos National Laboratory, Bioscience Division, Los Alamos, New Mexico, USA; 4 Biological Data Management and Technology Center, Lawrence Berkeley National Laboratory, Berkeley, California, USA; 5 Oak Ridge National Laboratory, Oak Ridge, Tennessee, USA; 6 HZI – Helmholtz Centre for Infection Research, Braunschweig, Germany; 7 University of California Davis Genome Center, Davis, California, USA; 8 Australian Centre for Ecogenomics, School of Chemistry and Molecular Biosciences, The University of Queensland, Brisbane, Australia

**Keywords:** strictly anaerobic, non-motile, Gram-negative, straight rod-shaped, halophilic, moderate alkaliphile, mesophilic, chemoorganotroph, *Halanaerobiaceae*, GEBA

## Abstract

*Halanaerobium praevalens* Zeikus *et al*. 1984 is the type species of the genus *Halanaerobium*, which in turn is the type genus of the family *Halanaerobiaceae*. The species is of interest because it is able to reduce a variety of nitro-substituted aromatic compounds at a high rate, and because of its ability to degrade organic pollutants. The strain is also of interest because it functions as a hydrolytic bacterium, fermenting complex organic matter and producing intermediary metabolites for other trophic groups such as sulfate-reducing and methanogenic bacteria. It is further reported as being involved in carbon removal in the Great Salt Lake, its source of isolation. This is the first completed genome sequence of a representative of the genus *Halanaerobium* and the second genome sequence from a type strain of the family *Halanaerobiaceae*. The 2,309,262 bp long genome with its 2,110 protein-coding and 70 RNA genes is a part of the *** G****enomic* *** E****ncyclopedia of* *** B****acteria and* *** A****rchaea * project.

## Introduction

Strain GSL^T^ (= DSM 2228 =ATCC 33744) is the type strain of the species *Halanaerobium praevalens*, which is the type species of its genus *Halanaerobium* [1]. Originally described as *Haloanaerobium* [2], the name was later changed to *Halanaerobium* to conform with rule 61 of the Bacteriological Code [3]. The genus currently consists of nine validly named species [4]. The genus name is derived from the Latinized Greek word *hals*; *halos* meaning *salt*, the Latinized Greek word *an-* meaning *not*, the Latinized Greek word *aer* meaning *air* and the Latinized Greek word *bios* meaning *life*, yielding the Neo-Latin word 'Halanaerobium' meaning 'salt organism which grows in the absence of air' [4]. The species epithet is derived from the Latin word 'praevalens' (very powerful, very strong, here prevalent) [4]. Strain GSL^T^ was isolated from the hypersaline surface sediments of Great Salt Lake, Utah, USA [2]. Further strains of *H. praevalens* have been isolated from canned salted Swedish fermented herrings referred to as Surströmming [5] and probably also from the Red Sea [6]. Other members of the genus have been isolated also from high salt environments distributed worldwide [7-10]. The enzymatic activities of the fatty acid synthetase complex and the *D*-BAPA (N’-benzoyl-arginine-*p-*nitroanilide)-ase of *H. praevalens* have been studied in more detail [11,12]. Here we present a summary classification and a set of features for *H. praevalens* strain GSL^T^, together with the description of the complete genome sequencing and annotation.

## Classification and features

A representative genomic 16S rRNA sequence of *H. praevalens* was compared using NCBI BLAST under default values (e.g., considering only the best 250 hits) with the most recent release of the Greengenes database [13] and the relative frequencies, weighted by BLAST scores, of taxa and keywords (reduced to their stem [14]) were determined. The five most frequent genera were *Halanaerobium* (81.9%), *Halothermothrix* (7.8%), *Halanaerobacter* (2.7%), *Acetohalobium* (2.3%) and *Natroniella* (1.9%). Regarding hits to sequences from other members of the genus, the average identity within HSPs (high-scoring segment pairs) was 97.8%, whereas the average coverage by HSPs was 96.3%. The species yielding the highest score was *Halanaerobium saccharolyticum*. (Note that the Greengenes database uses the INSDC (= EMBL/NCBI/DDBJ) annotation, which is not an authoritative source for nomenclature or classification.) The five most frequent keywords within the labels of environmental samples which yielded hits were 'microbi' (9.4%), 'hypersalin' (9.1%), 'mat' (8.6%), 'len, miniprim, new, view, world' (8.5%) and 'food' (3.4%). The single most frequent keyword within the labels of environmental samples which yielded hits of a higher score than the highest scoring species was 'hypersalin, len, mat, microbi, miniprim, new, view, world' (12.5%). These key words are in line with the ecology and the niche from where strains of *H. praevalens* have been isolated.

**Figure 1:**
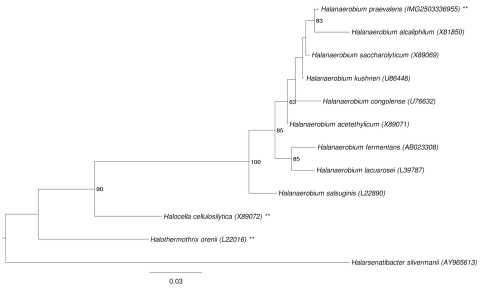
Phylogenetic tree highlighting the position of *H. praevalens* GSL^T^ relative to the other type strains within the family *Halanaerobiaceae*. The tree was inferred from 1,460 aligned characters [15,16] of the 16S rRNA gene sequence under the maximum likelihood criterion [17] and rooted in accordance with the current taxonomy. The branches are scaled in terms of the expected number of substitutions per site. Numbers to the right of bifurcations are support values from 1,000 bootstrap replicates [18] if larger than 60%. Lineages with type strain genome sequencing projects registered in GOLD [19] are labeled with an asterisk, published genomes with two asterisks [20].

Figure 1 shows the phylogenetic neighborhood of *H. praevalens* GSL^T^ in a 16S rRNA gene based tree. The sequences of the four 16S rRNA gene copies in the genome differ from each other by up to five nucleotides, and differ by up to five nucleotides from the previously published 16S rRNA gene sequence (AB022034).

**Figure 2:**
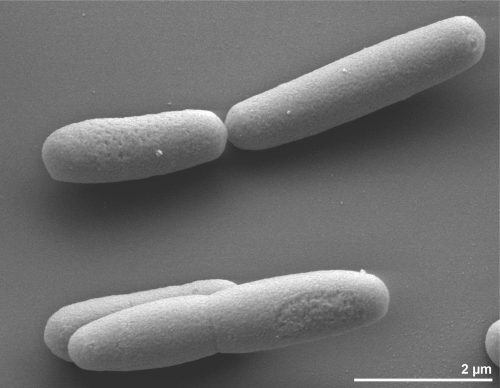
Scanning electron micrograph of *H. praevalens* GSL^T^

The cells of strain GSL^T^ are straight and rod-shaped (1 × 2.4 µm) (Figure 2) when grown to the mid-log phase at 37ºC on CS medium containing 12.5% NaCl and 0.5% glucose [2]. When grown at higher NaCl concentrations (> 20%) the cells appear granulated and shorter in length [2]. Single colonies were reported as white to translucent in color, 0.5-2.0 mm in diameter, and glistened, when grown on agar plates containing CS medium, 12.5% NaCl, and 0.5% glucose [2]. *H. praevalens* cells stain Gram-negative [2] and electron microscopy in thin section revealed architectural features typical of Gram-negative bacteria [2]. However, the positive *D*-BAPA-ase reaction [11] confirms its phylogenetic affiliation to the endospore-forming firmicutes (Table 1). In this respect, *H. praevalens* is able to hydrolyze only the D- but not the L- isomer of N’-benzoyl-arginine-*p-*nitroanilide (BAPA) [11]. The activity of *D*-BAPA-ase was highest at low NaCl concentration (100 mM) and completely inhibited at NaCl concentration equal or higher than 1.0 M (~12%) [11]. Strain GSL^T^ is described to be non-motile, although many flagellar genes have been identified in the genome (see below). Other isolates of *H. praevalens* were described as motile [5,6], as were other members of the genus [7,9,10], suggesting strain GSL^T^ is atypical with regard to motility. The organism is a strictly anaerobic chemoorganotroph [2]. It grows at NaCl concentrations between 2% and 30%, with optimal growth at approximately 13% [2]. The doubling time is 4 h at 12.5% NaCl and 7 h at 25% NaCl in complex CS medium [2]. The temperature range for growth ranges from 5ºC to 60ºC, with an optimum at 37ºC [2]. The pH range for growth is between 6.0 and 9.0, with an optimum at pH between 7.0 and 7.4 [2]. Strain GSL^T^ is able to utilize carbohydrates (including pectin and *N*-acetylglucosamine), amino acids, yeast extract, and trypticase; the two latter serving as carbon and energy sources on complex medium [2]. The fermentation of glucose yielded butyrate, acetate, propionate, H_2_, and CO_2_ as major products [2]. Also, fructose, D-mannose and maltose are utilized and methionine is transformed to methylmercaptan [2]. Penicillin, tetracycline, cycloserine, chloramphenicol (each at 100 µg/ml culture) or sodium azide (500 µg/ml) completely inhibit the growth of *H. praevalens* [2]. Strain GSL^T^ was also able to degrade nitro-substituted aromatic compounds such as nitrobenzene, *o*-nitrophenol, *m*-nitrophenol, *p*-nitrophenol, 2,4-dinitrophenol, and 2,4-dinitroaniline [34]. The fatty acid synthetase of *H. praevalens* is only slightly inhibited at 17.5% and was the first reported to be active in the presence of high salt concentrations [12]. *H. praevalens* was reported to be involved in carbon sequestration in the Great Salt Lake [35], since it is present in the sediments of this lake in high numbers (≥ 10^8^ cells/ml) [2,36]. *H. praevalens* regulates its internal osmotic pressure by the accumulation of salts (Na^+^, K^+^, Cl^-^) rather than by compatible solutes [36]. High concentrations of these salts were measured inside the cells, in sufficient concentration to be isotonic or hypertonic with the medium [37]. Thiosulfate reduction and rhodanese-like enzyme (thiosulfate:cyanide sulfur-transferase) activities also tested positive in strain GSL^T^ [8]. Early in 1987, Matheson *et al*. [38] established the primary structure of the ribosomal A-protein of the strain GSL^T^, which is the equivalent to the ribosomal protein L12 from *Escherichia coli*.

**Table 1:** Classification and general features of *H. praevalens* GSL^T^ according to the MIGS recommendations [21] and the NamesforLife database [22].

**MIGS ID**	**Property**	**Term**	**Evidence code**
	Current classification	Domain *Bacteria*	TAS [23]
		Phylum *Firmicutes*	TAS [24,25]
		Class *Clostridia*	TAS [26,27]
		Order *Halanaerobiales*	TAS [28-30]
		Family *Halanaerobiaceae*	TAS [1,31]
		Genus *Halanaerobium*	TAS [1-3,30]
		Species *Halanaerobium praevalens*	TAS [1,2]
		Type strain GSL	TAS [2]
	Gram stain	negative	TAS [2]
	Cell shape	straight rods	TAS [2]
	Motility	non-motile	TAS [2]
	Sporulation	none	TAS [2]
	Temperature range	above 5ºC and below 60ºC	TAS [2]
	Optimum temperature	37°C	TAS [2]
	Salinity	2%-30% NaCl, optimum at 13%	TAS [2]
MIGS-22	Oxygen requirement	strictly anaerobic	TAS [2]
	Carbon source	yeast extract, trypticase	TAS [2]
	Energy metabolism	chemoorganotroph	TAS [2]
MIGS-6	Habitat	saline environments	TAS [2]
MIGS-15	Biotic relationship	not reported	NAS
MIGS-14	Pathogenicity	not reported	NAS
	Biosafety level	1	TAS [32]
	Isolation	surface sediments of a saline lake	TAS [2]
MIGS-4	Geographic location	Great Salt Lake, Utah, USA	TAS [2]
MIGS-5	Sample collection time	between August 1979 and August 1980	TAS [2]
MIGS-4.1	Latitude	41.15	NAS
MIGS-4.2	Longitude	-112.67	NAS
MIGS-4.3	Depth	10 m bottom sediment	TAS [2]
MIGS-4.4	Altitude	1.755 m above sea level	NAS

Evidence codes - IDA: Inferred from Direct Assay (first time in publication); TAS: Traceable Author Statement (i.e., a direct report exists in the literature); NAS: Non-traceable Author Statement (i.e., not directly observed for the living, isolated sample, but based on a generally accepted property for the species, or anecdotal evidence). These evidence codes are from of the Gene Ontology project [33]. If the evidence code is IDA, the property was directly observed by one of the authors or an expert mentioned in the acknowledgements.

### Chemotaxonomy

When grown on CS medium, at 5% NaCl, more lipids are produced than at 25% NaCl (3.74% and 2.54% of the dry weight of the organism, respectively) [2]. At 5% NaCl, the fractions of glycolipids and phospholipids are 46.9% and 44.5% of the total lipids, respectively. At 25%, the proportion changes in favor of phospholipids (49.1%), whereas glycolipids decrease (43.0%) [2]. The glycolipids consist of a single component diacylglycerol derivative, while the phospholipids consist mainly of cardiolipin (CL), phosphatidyl glycerol (PG), and three minor unidentified constituents [2]. When grown on CS medium, at 5%, the major fatty acids are C_14:0_ (49.3%), C_16:1_ (31.3%) and C_16:0_ (11.4%). At 25%, these fractions change to 36.8%, 39%, and 22.7%, respectively. Similar though more detailed results on the fatty acid composition have been reported recently [6].

## Genome sequencing and annotation

### Genome project history

This organism was selected for sequencing on the basis of its phylogenetic position [39], and is part of the *** G****enomic* *** E****ncyclopedia of* *** B****acteria and* *** A****rchaea * project [40]. The genome project is deposited in the Genome On Line Database [19] and the complete genome sequence is deposited in GenBank. Sequencing, finishing and annotation were performed by the DOE Joint Genome Institute (JGI). A summary of the project information is shown in Table 2.

**Table 2:** Genome sequencing project information

**MIGS ID**	**Property**	**Term**
MIGS-31	Finishing quality	Finished
MIGS-28	Libraries used	Three genomic libraries: one 454 pyrosequence standard library, one 454 PE library (13 kb insert size), one Illumina library
MIGS-29	Sequencing platforms	Illumina GAii, 454 GS FLX Titanium
MIGS-31.2	Sequencing coverage	201.2 × Illumina; 174.2 × pyrosequence
MIGS-30	Assemblers	Newbler version 2.3, Velvet, phrap
MIGS-32	Gene calling method	Prodigal 1.4, GenePRIMP
	INSDC ID	CP002175
	Genbank Date of Release	October 21, 2010
	GOLD ID	Gc01415
	NCBI project ID	32591
	Database: IMG-GEBA	2503283011
MIGS-13	Source material identifier	DSM 2228
	Project relevance	Tree of Life, GEBA

### Growth conditions and DNA isolation

*H. praevalens* GSL^T^, DSM 2228, was grown anaerobically in DSMZ medium 210 (‘*Haloanaerobium*’ medium) [41] at 30-37°C. DNA was isolated from 0.5-1 g of cell paste using MasterPure Gram-positive DNA purification kit (Epicentre MGP04100) following the standard protocol as recommended by the manufacturer, with modification st/DL for cell lysis as described in Wu *et al*. [40]. DNA is available through the DNA Bank Network [42].

### Genome sequencing and assembly

The genome was sequenced using a combination of Illumina and 454 sequencing platforms. All general aspects of library construction and sequencing can be found at the JGI website [43]. Pyrosequencing reads were assembled using the Newbler assembler (Roche). The initial Newbler assembly, consisting of 85 contigs in 31 scaffolds, was converted into a phrap [44] assembly by making fake reads from the consensus, to collect the read pairs in the 454 paired end library. Illumina sequencing data (360 Mb) was assembled with Velvet [45] and the consensus sequences were shredded into 1.5 kb overlapped fake reads and assembled together with the 454 data. The 454 draft assembly was based on 401.6 Mb 454 draft data and all of the 454 paired end data. Newbler parameters are -consed -a 50 -l 350 -g -m -ml 20. The Phred/Phrap/Consed software package [44] was used for sequence assembly and quality assessment in the subsequent finishing process. After the shotgun stage, reads were assembled with parallel phrap (High Performance Software, LLC). Possible mis-assemblies were corrected with gapResolution [43], Dupfinisher [46], or sequencing cloned bridging PCR fragments with subcloning. Gaps between contigs were closed by editing in Consed, by PCR and by Bubble PCR primer walks (J.-F. Chang, unpublished). A total of 417 additional reactions and ten shatter libraries were necessary to close gaps and to raise the quality of the finished sequence. Illumina reads were also used to correct potential base errors and increase consensus quality using a software Polisher developed at JGI [47]. The error rate of the completed genome sequence is less than 1 in 100,000. Together, the combination of the Illumina and 454 sequencing platforms provided 375.4 × coverage of the genome. The final assembly contained 838,597 pyrosequence and 12,903,210 Illumina reads.

### Genome annotation

Genes were identified using Prodigal [48] as part of the Oak Ridge National Laboratory genome annotation pipeline, followed by a round of manual curation using the JGI GenePRIMP pipeline [49]. The predicted CDSs were translated and used to search the National Center for Biotechnology Information (NCBI) non-redundant database, UniProt, TIGR-Fam, Pfam, PRIAM, KEGG, COG, and InterPro databases. Additional gene prediction analysis and functional annotation was performed within the Integrated Microbial Genomes - Expert Review (IMG-ER) platform [50].

## Genome properties

**Figure 3:**
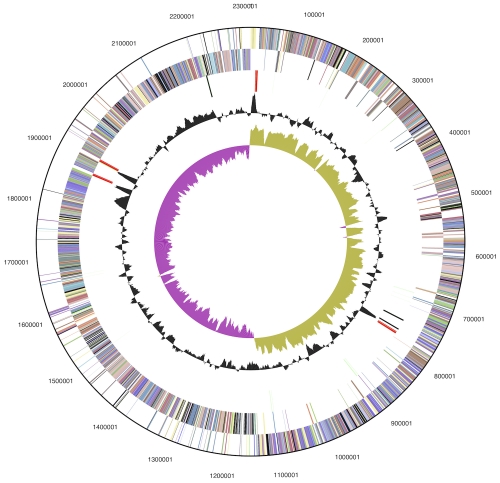
Graphical circular map of the chromosome. From outside to the center: Genes on forward strand (color by COG categories), Genes on reverse strand (color by COG categories), RNA genes (tRNAs green, rRNAs red, other RNAs black), GC content, GC skew.

The genome consists of a 2,309,262 bp long chromosome with a G+C content of 30.3% (Table 3 and Figure 3). Of the 2,180 genes predicted, 2,110 were protein-coding genes, and 70 RNAs; 42 pseudogenes were also identified. The majority of the protein-coding genes (77.7%) were assigned with a putative function while the remaining ones were annotated as hypothetical proteins. The distribution of genes into COGs functional categories is presented in Table 4.

**Table 3:** Genome Statistics

**Attribute**	**Value**	**% of Total**
Genome size (bp)	2,309,262	100.00%
DNA coding region (bp)	2,059,925	89.20%
DNA G+C content (bp)	699,559	30.29%
Number of replicons	1	
Extrachromosomal elements	0	
Total genes	2,180	100.00%
RNA genes	70	3.21%
rRNA operons	4	
Protein-coding genes	2,110	96.79%
Pseudo genes	42	1.93%
Genes with function prediction	1,694	77.71%
Genes in paralog clusters	310	14.22%
Genes assigned to COGs	1,760	80.73%
Genes assigned Pfam domains	1,860	85.32%
Genes with signal peptides	446	20.46%
Genes with transmembrane helices	582	26.70%
CRISPR repeats	1	

**Table 4:** Number of genes associated with the general COG functional categories

**Code**	**value**	**%age**	**Description**
J	133	6.9	Translation, ribosomal structure and biogenesis
A	0	0.0	RNA processing and modification
K	124	6.4	Transcription
L	115	6.0	Replication, recombination and repair
B	1	0.0	Chromatin structure and dynamics
D	24	1.2	Cell cycle control, cell division, chromosome partitioning
Y	0	0.0	Nuclear structure
V	18	0.9	Defense mechanisms
T	126	6.5	Signal transduction mechanisms
M	106	5.5	Cell wall/membrane/envelope biogenesis
N	76	3.9	Cell motility
Z	0	0.0	Cytoskeleton
W	0	0.0	Extracellular structures
U	44	2.3	Intracellular trafficking, secretion, and vesicular transport
O	66	3.4	Posttranslational modification, protein turnover, chaperones
C	123	6.4	Energy production and conversion
G	150	7.8	Carbohydrate transport and metabolism
E	136	7.0	Amino acid transport and metabolism
F	74	3.8	Nucleotide transport and metabolism
H	80	4.1	Coenzyme transport and metabolism
I	45	2.3	Lipid transport and metabolism
P	121	6.3	Inorganic ion transport and metabolism
Q	18	0.9	Secondary metabolites biosynthesis, transport and catabolism
R	186	9.6	General function prediction only
S	168	8.7	Function unknown
-	420	19.3	Not in COGs
